# Electrophoretic Deposition and Characterization of the Doped BaCeO_3_ Barrier Layers on a Supporting Ce_0.8_Sm_0.2_O_1.9_ Solid-State Electrolyte

**DOI:** 10.3390/membranes12030308

**Published:** 2022-03-09

**Authors:** Elena Kalinina, Kirill Shubin, Elena Pikalova

**Affiliations:** 1 Laboratory of Complex Electrophysic Investigations, Institute of Electrophysics, Ural Branch of the Russian Academy of Sciences, 620016 Yekaterinburg, Russia; e.pikalova@list.ru; 2 Department of Physical and Inorganic Chemistry, Institute of Natural Sciences and Mathematics, Ural Federal University, 620002 Yekaterinburg, Russia; 3 Laboratory of Solid Oxide Fuel Cells, Institute of High Temperature Electrochemistry, Ural Branch of the Russian Academy of Sciences, 620137 Yekaterinburg, Russia; k.shubin@ihte.uran.ru; 4 Department of Environmental Economics, Institute of Economics and Management, Ural Federal University, 620002 Yekaterinburg, Russia

**Keywords:** electrophoretic deposition, MIEC electrolyte, barrier layer, doped BaCeO_3_, deposition kinetics, conducting polymer

## Abstract

In this study, the technology of electrophoretic deposition (EPD) micrometer barrier layers based on a BaCe_0.8_Sm_0.19_Cu_0.1_O_3_ (BCSCuO) protonic conductor on dense carrying Ce_0.8_Sm_0.2_O_1.9_ (SDC) solid-state electrolyte substrates is developed. Methods for creating conductive sublayers on non-conductive SDC substrates under EPD conditions, such as the synthesis of a conductive polypyrrole (PPy) layer and deposition of a layer of finely dispersed platinum from a suspension of its powder in isopropanol, are proposed. The kinetics of disaggregation, disperse composition, electrokinetic potential, and the effect of adding iodine to the BCSCuO suspension on these parameters as factors determining the preparation of stable suspensions and successful EPD processes are explored. Button cells based on a carrying SDC electrolyte of 550 μm in thickness with BCSCuO layers (8–35 μm) on the anode, cathode, and anode/cathode side, and Pt electrodes are electrochemically tested. It was found that the effect of blocking the electronic current in the SDC substrate under *OCV* conditions was maximal for the cells with barrier layers deposited on the anode side. The technology developed in this study can be used to fabricate solid oxide fuel cells with doped CeO_2_ electrolyte membranes characterized by mixed ionic–electronic conductivity (MIEC) under reducing atmospheres.

## 1. Introduction

Barrier layers are widely used in the technology of solid oxide fuel cells (SOFCs) to prevent the interaction of the individual functional layers of SOFCs, to protect electrode layers from phase decomposition and poisoning, and to increase the SOFC energy efficiency by blocking the electron current in devices with electrolyte membranes possessing mixed conductivity (MIECs) [1,2,3,4,5]. Prospective cathode materials with high mixed electronic–ionic conductivity with a perovskite or perovskite-like structure, such as lanthanum–strontium manganites and cobaltite–ferrites, layered nickelates of rare earth metals (La, Pr, or Nd), and various double perovskites are incompatible with conventional electrolyte materials based on ZrO_2_ or LaGaO_3_ due to the diffusion of cations and the resulting chemical interaction at the electrode–electrolyte interface which causes an increase in polarization resistance and can also lead to the delamination of the cathode layer [6,7,8]. This problem is solved by the formation of barrier layers between the main electrolyte layer (such as, for example, yttria-doped zirconia (YSZ)) and the cathode. The most commonly used materials for such barrier layers are Gd, Sm, or Y-doped CeO_2_ solid-state electrolytes, which have high ionic conductivity and low interaction with perovskite materials; thus, their use in conventional YSZ-based SOFCs has the effect of both enhancing the cell performance and extending its lifetime remarkably [9,10]. Alternatively, materials based on doped CeO_2_ are promising electrolyte materials for intermediate and low-temperature (IT and LT) SOFCs operating in the range of 450–750 °C [2,11,12]. However, at temperatures above 600 °C, CeO_2_-based MIEC electrolytes are characterized by the appearance of significant electronic conductivity at low partial pressures of oxygen. Consequently, in the SOFC operating mode, an internal leakage current occurs, which causes a reduction in the open circuit value (*OCV*) and cell-specific power [2,13]. In order to block the electron leakage current in SOFC cells based on the MIEC electrolyte, Y or Sc-doped ZrO_2_ electrolytes, which are unipolar ionic conductors across a wide range of oxygen partial pressure, can be used as barrier materials. However, there is a problem associated with decreasing their ionic conductivity and increasing the activation energy in the IT- and LT-ranges. There is also an undesirable chemical interaction with CeO_2_-based electrolytes when sintering the barrier layers at temperatures above 1250 °C in the manufacturing process [14]. Moreover, due to insufficient thermomechanical compatibility (the thermal expansion coefficient (*TEC*) values of YSZ and Sm-doped ceria are 10 and 12 × 10^−6^ K^−1^, respectively), in the case of a dense barrier layer, its delamination is quite possible.

Doped BaCeO_3_ solid-state electrolytes with a perovskite structure are promising materials for barrier layers, being compatible with electrolytes based on CeO_2_ in terms of their thermomechanical properties [15]. They are characterized by a high level of co-ionic (proton and oxygen ion) conductivity in the IT and LT ranges with a low activation energy [16] and show no chemical interaction with CeO_2_ electrolytes, even under one-pot synthesis using a nitrate combustion procedure [17,18]. When used as a cathode-side-blocking layer, the appearance of electronic p-type conductivity under oxidizing conditions in the doped BaCeO_3_ can be favorable for current leakage blocking in CeO_2_-based cells [19,20].

Various methods are used for the deposition of barrier layers in SOFC technology [21,22]: ceramic methods, such as screen-printing [23] and tape calendering [24,25]; vacuum deposition technologies, e.g., magnetron sputtering [26,27], pulsed laser deposition [10,28], and physical vapor deposition (PVD) [29]; aerosol-spraying methods under atmospheric [30] and reduced pressures [31]; and colloidal and solution technologies—electrophoretic deposition [32,33], dip-coating and sol-gel [34,35], suspension centrifugation [36] etc. One of the flexible, easy-to-implement, and cheap technologies is electrophoretic deposition (EPD), which does not require high-tech equipment and allows the deposition of coatings at room temperature in ambient air with a sufficiently high deposition rate of ~1–10 μm per 1 min [37]. Compared to the most widely-used screen-printing technique, the EPD method is suitable for the fabrication of highly dense films due to the lower content, or even absence, of organic additives inherent in the process. This allows obtaining films with green density similar to that of cold-pressed compacts [38]. In contrast to the majority of the ceramic deposition methods, EPD can be applied for the deposition of films on substrates of different shapes (planar, tubular, coned, etc.).

A necessary condition for the implementation of the EPD process is the presence of sufficient surface conductivity for the substrate used for the deposition. EPD can be successfully carried out on highly conductive cathode substrates (conductivity ˃ 10 S/cm) [39,40], while deposition on NiO–cermet anodes or solid-state electrolyte substrates, which possess extremally low conductivity under EPD conditions, requires additional measures to create surface conducting layers or volume conducting paths in the case of porous substrates [41,42]. An important issue for EPD implementation is the preparation of aggregatively stable suspensions through the choice of suitable dispersion media and additives to ensure the reproducibility of the developed technology [43]. Another important issue is the choice of proper sintering conditions to prevent the deformation of the cell comprising heterogeneous layers and their delamination.

In this paper, the studies of our scientific group on the deposition of micrometer barrier layers based on a BaCe_0.8_Sm_0.19_Cu_0.1_O_3_ (BCSCuO) co-ionic conductor on dense carrying Ce_0.8_Sm_0.2_O_1.9_ (SDC) solid-state electrolyte substrates are presented. The Sm-doped BaCeO_3_ electrolyte was selected as the most chemically compatible electrolyte with SDC [17]. Cu-doping was applied to increase the sintering abilities of this material, making it possible to obtain fully dense films at a lower temperature than the substrate sintering temperature in order to avoid its curvature. To fabricate the BCSCuO barrier layers by EPD on the SDC substrates, which are non-conductive under EPD conditions, methods for creating conductive sublayers, such as the surface synthesis of a conductive polypyrrole (PPy) layer or the deposition of a layer of finely dispersed platinum from a suspension of its powder in isopropanol, were developed. Additionally, the kinetics of disaggregation, disperse composition, electrokinetic potential, as well as the effect of the iodine introduction into the suspension on these parameters, which ensure the preparation of stable suspensions of the microsized BCSCuO powder for successful EPD implementation, were investigated. The kinetics of the EPD process were studied and a mode was chosen that provided the required thicknesses of the BCSCuO barrier layers. To clarify the effect of the deposited BCSCuO barrier layers on the electronic current blocking, button cells based on the carrying SDC electrolyte of 550 μm in thickness with the barrier layers on the anode, cathode, or anode/cathode sides and Pt electrodes were formed and electrochemically tested. The effect of the barrier layers on the electronic leakage current was demonstrated. The technology developed in this study for the deposition of barrier layers can be used to fabricate solid oxide fuel cells with MIEC electrolyte membranes based on doped CeO_2_.

## 2. Materials and Methods

### 2.1. Synthesis and Characterization of the Electrolytes

Synthesis of the BaCe_0.8_Sm_0.19_Cu_0.01_O_3_ (BCSCuO) electrolyte was carried out by a citrate–nitrate combustion technique using BaCO_3_ (99.2 wt.%), Ce(NO_3_)_3_·6H_2_O (99.9 wt.%), Sm(NO_3_)_3_·6H_2_O (99.0 wt.%), and CuO (99.0 wt.%) as the starting chemicals. CuO additive in a minimum amount of 1 mol. % was added to increase the sinterability of BCSO without the deterioration of its electrical properties [44]. CuO and BaCO_3_ were dissolved in a minimum amount of HNO_3_ to obtain the corresponding nitrates. Citric acid and glycerin were added to the starting materials taken in a stoichiometric amount in the following molar ratio—sum of metal cations:citric acid:glycerol = 1:0.5:1.5. A small amount of distilled water was added to the resulting mixture, which was then heated to 80 °C and kept at this temperature until the solid components were completely dissolved. Further, a 10% ammonia solution was added to fix the pH value at 6–7. The resulting solution was evaporated at a temperature of 200 °C before the self-ignition process started, resulting in the formation of a highly dispersed powder. The obtained powder was ground in an agate mortar in an isopropyl alcohol medium for 40 min. Next, a two-stage firing of the powder was carried out at temperatures of 1050 °C (5 h) and 1150 °C (5 h) with a heating and cooling rate of 5 °C/min with intermediate grinding in the agate mortar. The resulting powder was milled in an isopropyl alcohol medium for 3 h in a plastic drum with tetragonal zirconia milling bodies using a Pulverisette 7 planetary mill (Retsch, Haan, Germany) at a rate of 250 rpm. The specific surface area, *S_BET_*, of the milled powder, characterized using a BET method by means of a SORBI N 4.1 instrument (Meta, Moscow, Russia), was equal to 2.77 ± 0.05 m^2^/g. For the electroconductivity characterization, the BCSCuO powder was pressed under 250 MPa into a green bar sample of 25 × 5 × 5 mm in size and sintered at 1450 °C (5 h) with a heating and cooling rate of 2 °C/min.

The synthesis of the Ce_0.8_Sm_0.2_O_1.9_ (SDC) electrolyte was carried out by a solid-state reaction technique using Ce_2_(CO_3_)_3_ (99.99 wt.%) and Sm_2_O_3_ (99.9 wt.%) as the starting chemicals. The starting materials taken in a stoichiometric amount were mixed for 1 h in a mill at 200 rpm in an isopropyl alcohol medium. The dried powders were calcined in a closed zirconia crucible at 950 °C (10 h) and 1150 °C (10 h) with a heating and cooling rate of 5 °C/min with intermediate grinding in the agate mortar. The resulting powder was milled in an isopropyl alcohol medium for 3 h at 250 rpm down to *S_BET_* = 2.50 ± 0.05 m^2^/g. The SDC powder was pressed into a bar sample for the electroconductivity study of 25 × 5 × 5 mm in size and into disks with 15 mm in diameter and 1 mm in thickness for the substrates and sintered at 1600 °C (3 h) with a heating and cooling rate of 2 °C/min.

The X-ray diffraction analysis of the obtained samples was carried out using an XRD-7000 diffractometer (Shimadzu, Kyoto, Japan) under CuKα radiation and in an angle range of 2θ = 25–80° in the shared access center of the Institute of Metallurgy, UB RAS (Yekaterinburg, Russia). Data processing and phase identification were performed using the PDF-4 database (ICDD, Newtown Square, PA, USA, Release 2018). The study of the powder morphology was performed using a JSM-6390 LA scanning electron microscope (JEOL, Tokyo, Japan).

The electroconductivity studies were performed by a four-probe *dc* current method using the bar-shaped samples. Pt wires of 0.2 mm in diameter were used as the current and voltage probes. They were attached to the sample, and the place of the contact was covered with Pt paste and sintered at 900 °C for 1 h to ensure good adherence. Measurements were performed at 500–800 °C in wet air (3% H_2_O, 7 L/h flow) and under an atmosphere with the controlled partial pressure of oxygen in the range of 0.21–10^−24^ atm at 600, 700, and 800 °C. The oxygen partial pressure in the cell with the closed volume was established using a Zirconia-M microprocessor for automatic temperature and partial pressure regulation and data recording (Ural Federal University, Yekaterinburg, Russia), comprising an electrochemical pump and a sensor made on the base of ZrO_2_–Y_2_O_3_ ceramic tube.

### 2.2. Preparation and Characterization of BCSCuO Suspensions for EPD

A suspension based on the BCSCuO powder with a concentration of 10 g/L in a mixed dispersion medium of isopropanol/acetylacetone (70/30 vol.%) was prepared by treatment in an UZV-13/150-TH ultrasonic bath (Realtek, St. Petersburg, Russia) at 210 W (at the operating frequency of 22 kHz) for 5–125 min (at 25 °C). The temperature in the bath was maintained at a given level by water exchange. Molecular iodine was added to the prepared suspension in an amount of 0.4 g/L. The electrokinetic zeta potential and pH of the suspension were measured by an electroacoustic method using a DT-300 analyzer (Dispersion Technology, Lakewood, NJ, USA). For the study by a method of dynamic light scattering, the BCSCuO suspension with a concentration of 1 g/L was prepared using the same ultrasonic treatment (UST) procedure. The particle size distribution and disperse composition in the low-concentration suspension were studied using a ZetaPlus particle size analyzer (Brookhaven, GA, USA).

### 2.3. Electrophoretic Deposition and Characterization of BCSCuO Films

Electrophoretic deposition was performed on the SDC substrates ground down to a thickness of 550 μm using diamond polishing disks followed by cleaning in an ultrasonic bath for 5 min and calcination at 600 °C for 1 h. The EPD modes during deposition were controlled using a specialized laboratory setup (IEP UB RAS, Yekaterinburg, Russia). The cathodic EPD of the BCSCuO layers was carried out in the constant voltage mode. During the EPD, the SDC substrate with a surface area of approximately 1.2 cm^2^ was placed on the cathode electrode in the EPD cell. The counter electrode was a stainless-steel disk with an area of 1 cm^2^ situated at a distance of 1 cm from the cathode.

The study of the surface and cross-sections of the deposited films was carried out in the BSE (back-scattered electron) and SE (secondary electron) modes using a JSM-6390 LA scanning electron microscope (JEOL, Tokyo, Japan) equipped with a system for energy-dispersion X-ray microanalysis (EDX). The morphology of the surface of the thin film layers at the intermediate deposition stages was studied using an VS-520 optical microscope (STAT, Yekaterinburg, Russia). The thickness of the as-deposited BCSCuO films was estimated from the deposition weight, the film surface area, and the theoretical density of BCSCuO, calculated using the XRD data. The actual thickness of the deposited and sintered films was determined from the corresponding cross-section SEM images.

### 2.4. Electrochemical Characterization of SDC Substrates with Deposited BCSCuO Films under Open-Circuit Conditions

The electrochemical study was performed using a laboratory setup comprising a B2901A precision source/measure unit (Keysight, Colorado Springs, CO, USA) and an RTM3004 oscillograph (Rohde&Schwarz, Munich, Germany) operated by a program written in LabVIEW and a Parstat 3000A potentiostat/galvanostat (Ametek Scientific Instruments, Oak Ridge, TN, USA). To prepare the samples for the electrochemical study, the BCSCuO barrier layers were deposited on one or both sides of the SDC electrolyte disks (relative density of 97–98% and 550 μm in thickness) using polypyrrole or Pt conducting surface coatings and sintered at 1450 °C for 5 h. Then, platinum electrodes with an effective area of 0.204 cm^2^ were brushed symmetrically on the opposite sides of the disks and sintered at 900 °C for 2 h. The activation of the platinum electrodes was performed by the infiltration of solutions of Ce and Pr nitrates, as described elsewhere [45]. The sample was installed in the measuring tubular cell with an internal air electrode and an external hydrogen electrode. The sample was fixed on the top of a YSZ ring using Aremco Ceramabond™ 571 (Aremco Products Inc., Valley Cottage, NY, USA) and then adhered tightly to the top of the measuring cell using a high-temperature sealant (at 930 °C). First, the impedance measurements were taken using a Parstat 3000A at an applied alternating signal of 30 mV at 30 points per decade in the current range of 200 mA at 800, 700, and 600 °C with both Pt electrodes blown with humidified air (3% H_2_O, 7.5 L/h flow rate). Then, at 600 °C, the oxidizing atmosphere in the anode channel (outside the tube) was gradually replaced with humidified hydrogen (3% H_2_O, 5 L/h flow rate). The *OCV* measured by the electrochemical sensor of the measuring cell (the cell description is given in [45]) was 1127 mV, indicating good settling strength of the sample to be measured. Further, measurements were carried out with the recording of the *OCV* values on the sample and the impedance spectrum under *OCV* conditions at 600 °C, 700 °C, and 800 °C. The impedance spectra were analyzed using ZView 3.4 software.

## 3. Results

### 3.1. Characteristics of the SDC and BCSCu Electrolyte Materials

**Figure 1:**
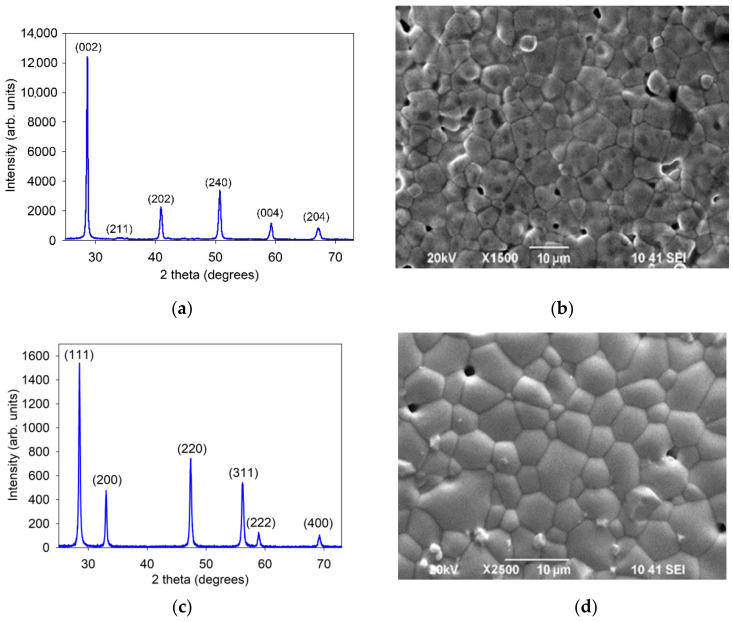
Characterization of the BCSCuO and SDC electrolytes: (**a**,**c**) XRD patterns of the BCSCuO and SDC powders; (**b**,**d**) Surface SEM images of the BCSCuO and SDC compact samples sintered at 1450 °C for 5 h and at 1600 °C for 3 h, respectively.

According to the XRD data (Figure 1a), the BCSCuO powder was single-phase and characterized by an orthorombic perovskite-type structure with a space group of Pnma (62) and lattice parameters of *a* = 6.2305(9) Å, *b* = 8.8010(14) Å, and *c* = 6.2268(11) Å (Figure 1c), close to those presented in literature [17,44,46]. After sintering at 1450 °C, the BCSCuO sample had a relative density of 95% with an average grain size, evaluated using the certified program based on the principles described in [47], equal to 7.8 μm (Figure 1b). The SDC compact sample had a relative density of 97% with an average grain size of 8 μm (Figure 1d). It was single-phase and had a cubic fluorite-type structure with an Fm–3m (225) space group and a lattice parameter of *a* = 5.4305(1) Å (Figure 1c).

According to the literature data, Cu-doping in a small amount allows dense BCSCuO electrolyte samples and films to be obtained at relatively low temperatures, while sintering the additive-free BCSO samples requires a sintering temperature as high as 1600 °C [47]. Increasing the sinterability of the materials used for the barrier layers by means of the overstoihiometric introduction of transition metals was also used in a number of studies [32,48,49]. These methods allow obtaining dense barrier layers from micro-sized powders using techniques that require high-temperature sintering steps, such as EPD or screen-printing.

**Figure 2:**
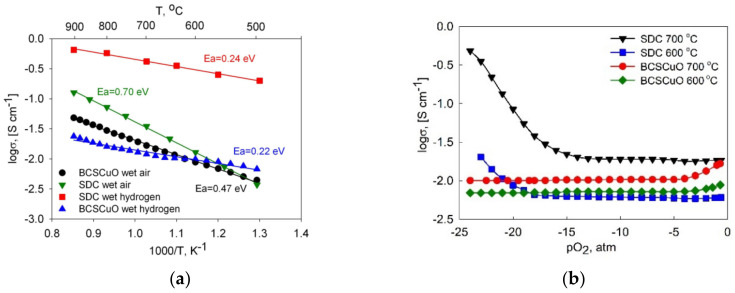
Electrical properties of BCSCuO and SDC compacts: (**a**) Conductivity temperature dependences in wet air and wet hydrogen; (**b**) Conductivity dependences on *pO*_2_ at 600 °C and 700 °C.

The temperature dependences of the total conductivity of the SDC and BCSCuO compact samples (in the Arrhenius coordinates) are presented in Figure 2a. The graph shows that the conductivity of the SDC sample in air in the high-temperature range (T > 600 °C) is higher than that of the BCSCuO sample, while in the low-temperature range, they become almost equal and even show an opposite trend. This can be explained by the lower activation energy of the total conductivity of the BaCeO_3_-based material, related to the presence of proton conductivity in the humidified air atmosphere, which becomes dominant at relatively low temperatures [16]. In hydrogen, the total conductivity of the SDC sample was much higher than that of the BCSCuO sample due to the appearance of the predominant electronic conductivity in the CeO_2_-based material caused by partial reduction of Ce^4+^→ Ce^3+^ under reducing conditions [2]. The level of conductivity and the calculated activation energies for the total conductivity in air and in hydrogen (shown in Figure 2a) are in good agreement with literature data for both the Sm-doped ceria [17,50,51] and for Sm and Sm/Cu-doped BaCeO_3_ [17,44,46].

Figure 2b represents the results of the conductivity study on the oxygen partial pressure (*pO*_2_). There are continuous flat regions on the dependences, corresponding to the electrolytic domain, where the electrolytes are predominantly ionic conductors. Increasing the conductivity of BCSCuO at high *pO*_2_ values and SDC at low *pO*_2_ values demonstrates the presence of the electronic conductivity of *p*- and *n*-types, respectively. At high dopant concentrations (20 mol. % in this study), the ionic conductivity, *σ_i_*, was defined mainly by the concentration of the dopant and did not depend on *pO*_2_ [2,16,17]. In this case, the partial p- and n-type electronic conductivities, *σ_p_* and *σ_n_*, could be evaluated by a difference method [52] by the subtraction of the ionic conductivity value (the value of total conductivity in the electrolytic domain) from the total conductivity values at the given *pO*_2_. The values calculated at 0.21 atm and 10^−22^ atm are shown in Table 1. Under the highly reducing atmosphere, the value of the n-type electronic conductivity for the SDC electrolyte was higher than that of the ionic conductivity, even at a temperature of 600 °C. The electrolyte domain boundary values (*pO*_2_ values at which the ionic and electronic conductivity are equal) for SDC at 600 °C and 700 °C were 10^−21.3^ atm and 10^−17.9^ atm, while BCSCuO behaved as a pure ionic conductor under reducing conditions.

The study of the electrical properties clearly demonstrated that the BCSCuO electrolyte, having predominantly ionic (protonic and oxygen ionic) conductivity in reducing conditions, may be an effective anode barrier layer for the SDC electrolyte. The effectiveness of a blocking layer is defined by its thickness and density, as well as its compatibility with a contacting electrode. Moreover, by varying the thickness of the barrier layer based on the doped BaCeO_3_ situated on the cathode site, it is possible to obtain a sufficient blocking effect as well, as was demonstrated theoretically by Wang et al. [20]. The authors investigated model cells with proton-conducting barrier layers of 19 μm in thickness deposited on the anode or cathode side of the SDC film of 11 μm in thickness. They also demonstrated that the barrier layer/main electrolyte thickness ratio can be optimized to maximize the electrochemical performance by balancing the *OCV* value and the level of the ohmic polarization losses. Habayashi et al. [53] experimentally established an optimal thickness for a BCS layer grown on an SDC electrolyte substrate of 500 μm in thickness via the solid-state reaction of 13 μm as being enough to successfully block the electronic leakage current. Thus, in this study, we chose the required thickness of the film for the deposition by the EPD method equal to be approximately 15–20 μm.

### 3.2. Preparation and Study of the Fractional Composition of the Base Suspension of the BCSCuO Electrolyte Powder for EPD

**Figure 3:**
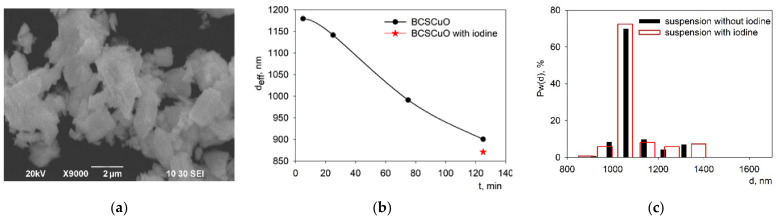
(**a**) Morphology of the initial BCSCuO powder for the suspension preparation; (**b**) Dependence of the effective hydrodynamic diameter of aggregates, *d_eff_*, on the UST time; (**c**) Fractional composition of the BCSCuO suspension with/without iodine.

The morphology of the initial BCSCuO powder used for the suspension preparation is presented in Figure 3a. The microsized BCSCuO powder consisted mainly of large plate-shaped particles of 1–4 µm in size, as well as of smaller submicron particles. A suspension based on the BCSCuO powder with a concentration of 1 g/L in a mixed dispersion medium of isopropanol/acetylacetone (70/30 vol.%) was prepared by UST for 5–125 min (under a constant temperature of 25 °C). According to the data obtained using the method of dynamic light scattering, the effective hydrodynamic diameter of the aggregates, *d_eff_*, in the suspension as a function of the time of UST decreased from the initial value of 1179 nm to the final value of 900 nm at the UST time of 125 min (Figure 3b).

The preliminary experiments on the EPD from the suspension with a solid loading concentration of 10 g/L showed that deposition from the base suspension did not occur. Thus, the suspension was modified by the addition of iodine. The value of *d_eff_* in the suspension with the addition of 0.4 g/L of iodine after 125 min of UST was 871 nm. The results of determining the fractional composition of the BCSCuO suspensions without and with the addition of iodine are shown in Figure 3c, where Pw(d) is the weight fraction of aggregates with size d. After ultrasonication for 125 min, the size distribution of aggregates was monomodal: the average size of the aggregates of the main fraction and their proportion were 1058 nm and 70%, respectively. The addition of iodine to the suspension had no pronounced effect on its fractional composition. The value of the zeta potential, ζ, in the suspension of the BCSCuO powder, measured by the electroacoustic method, was +11 mV (at pH = 4.3). The addition of iodine had no effect on the ζ value, while the pH shifted to a more acidic value and amounted to 3.7. Nevertheless, the use of iodine turned out to be necessary for the implementation of the EPD process. The use of iodine as an effective dispersant for EPD suspensions was demonstrated in a number of studies [54,55,56].

### 3.3. EPD from the Suspension of BCSCuO Powder on a Model Substrate (Ni-Foil): Determination of Optimal Deposition Modes

**Figure 4:**
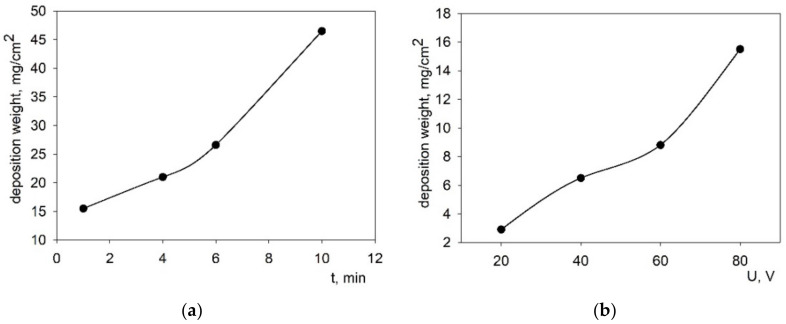
Growth of the deposited weight during the EPD from the suspension of 10 g/L of the BCSCuO powder with iodine (0.4 g/L): (**a**) as a function of time at a constant voltage of 80 V; (**b**) as a function of voltage at a constant deposition time of 1 min.

Studies of the EPD modes from the stabilized suspension of the BCSCuO powder (10 g/L) were carried out on a model electrode (Ni foil) to obtain continuous BCSCuO films of the required thickness in the range of 15–20 μm without cracks and pores. The dependences of the deposited weight on time were obtained at a fixed voltage of 80 V (Figure 4a) and at the voltage in the range of 20–80 V at a fixed deposition time of 1 min (Figure 4b) in a mixed dispersion medium of isopropanol/acetylacetone (70/30 vol.%) with the addition of iodine at a concentration of 0.4 g/L. As can be seen from Figure 4, there was a slight non-linearity in the dependence of the deposited weight on time and on voltage. As shown by preliminary experiments on the Ni-foil, a deposition time of 1–3 min at the voltage of 80 V was required to obtain a BCSCuO coating with the required thickness of 15–20 µm, calculated considering the deposited weight and the BCSCuO theoretical density equal to 6.33 r/cm^3^.

**Figure 5:**
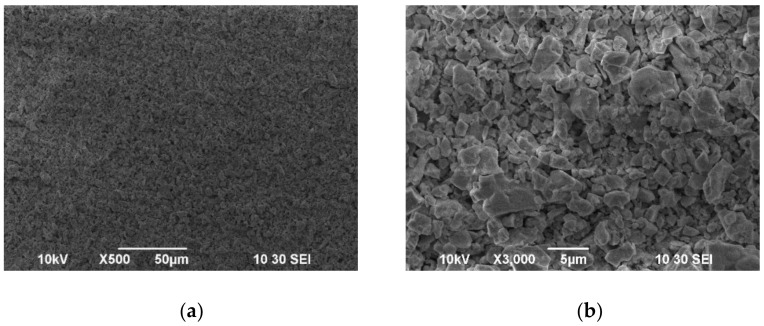
SEM images of the surface of the green BCSCuO coating on the model Ni foil electrode during the EDP from the suspension of 10 g/L of the BCSCuO powder with 0.4 g/L iodine: (**a**) ×500; (**b**) ×3000.

A test sample of the BCSCuO coating on the Ni foil substrate was obtained in the EPD mode at a constant voltage of 80 V for 2.5 min (Figure 5). It can be seen that the unsintered BCSCuO coating was continuous, crack-free, and consisted of large irregularly shaped particles of 1–4 µm in size, which corresponded to the morphology of the original powder (Figure 3a). The preliminary mode (80 V, 2.5 min) was subsequently used to form the BCSCuO barrier layers on the SDC electrolyte substrates with conductive layers of finely dispersed platinum or polypyrrole (PPy), as described in the next section.

### 3.4. EPD of the BCSCuO Layer on the SDC Substrate with a Predeposited Sublayer of Finely Dispersed Platinum

**Figure 6:**
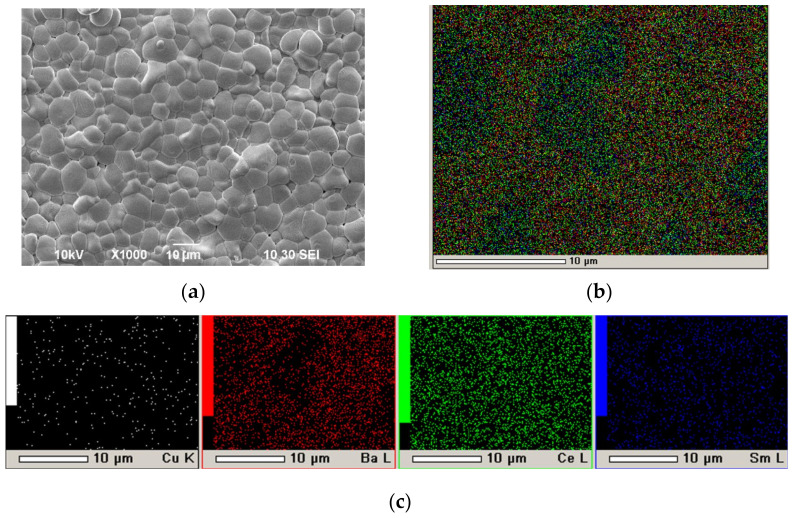
The surface of the BCSCuO electrolyte layer after the final sintering at 1500 °C for 3 h on the dense SDC substrate with the platinum sublayer (Sample SDC-Pt-BCSCuO): (**a**) SEM image; (**b**) Integrated map of the element distribution; (**c**) Maps of the individual elements.

In our recent work [57], we proposed a method for the successful EPD of electrolyte films on a non-conductive NiO–SDC anode substrate by creating on its surface a conductive sublayer by dropping the suspension of finely dispersed platinum (average particle size of 2 µm) with a pipette, followed by drying and annealing at 900 °C for 1 h. In the present work, this method was used to obtain a conducting Pt sublayer with a specific weight of 9.3 mg/cm^2^ on the dense SDC substrate (550 μm) for the following EPD of the BCSCuO layer. The formation of the BCSCuO coating (Sample SDC-Pt-BCSCuO) was carried out in three deposition–sintering cycles: first cycle, 3.5 min (80 V), thickness 8.5 µm, sintering at 1200 °C, 5 h; second cycle—7.5 min (80 V), thickness 5.5 µm, sintering at 1200 °C, 5 h; third cycle—4.2 min (80 V), thickness 3.6 µm, sintering at 1500 °C, 3 h. As a result of the final sintering, a BCSCuO coating with a thickness of 17.6 µm was obtained. The surface of the BCSCuO coating is shown in Figure 6a. It can be seen that a continuous sintered layer with an average grain size of 10 µm was formed. According to the EDX analysis (Figure 6b,c), the coating composition corresponded to the nominal composition for the BCSCuO electrolyte. It should be noted that, during the deposition on the Pt sublayer, including that with the use of the intermediate sintering stages, the deposition rate decreased in contrast to EPD on the Ni foil, which was probably caused by the porous nature of the Pt sublayer. Nevertheless, the Pt sublayer was heat-resistant and retained its conductivity at the sintering temperature, which allowed for multiple deposition–sintering cycles with a single initial deposition of the conductive sublayer.

### 3.5. Formation of a Conductive Polypyrrole Sublayer on the Surface of the Dense SDC Electrolyte Substrates

Various methods are provided in the literature for the synthesis of conductive polypyrrole polymers [58]. For example, Sakthivel and Boopathi synthesized the PPy powder in a solution of pyrrole (1 M) and ammonium persulfate (NH_4_)_2_S_2_O_8_ [59]. After completion of the synthesis, the resulting powder was filtered off and washed with distilled water, after which it was dried for 2 days. The PPy powder was dissolved in m-cresol for 4–5 days under constant stirring. A PPy film was deposited on a non-conductive substrate by a spin-coating method at 3000 rpm, and the resulting conductive polypyrrole coating was dried for 1 h. The drawback of this method is the use of the toxic organic solvent m-cresol. Moreover, the use of m-cresol causes difficulties in the formation of films by spin-coating—due to the continuous evaporation of the solvent, there is a risk of obtaining heterogeneous and uneven coatings. The direct synthesis of a PPy film on a YSZ electrolyte substrate for the following EPD of the Gd-doped ceria was presented by Hu et al. [32]. The substrate was immersed in a mixture of (NH_4_)_2_S_2_O_8_ and 2,6-disodium salt of naphthalenedisulfonic acid with a concentration of each of the reagents of 0.0067 M in distilled water, and pyrrole (monomer) was added to the solution up to the concentration of 0.14 M. Synthesis was carried out for 6 h at 0 °C. The resulting coating was dried at room temperature. Suzuki et al. [60] synthesized PPy films in a mixed aqueous solution using 0.01 M (NH_4_)_2_S_2_O_8_ as an oxidizing agent and 0.01 M of naphthalene disulfonic acid disodium salt 2,6-disulfonic acid as a dopant. The substrate composed of the La_0.8_Sr_0.2_Ga_0.8_Mg_0.2_O_3–__δ_ (LGSM) electrolyte was immersed in this solution, and then pyrrole was added up to the concentration of 0.001 M. Synthesis was carried out at 0 °C for 12 h, followed by drying at room temperature.

**Figure 7:**
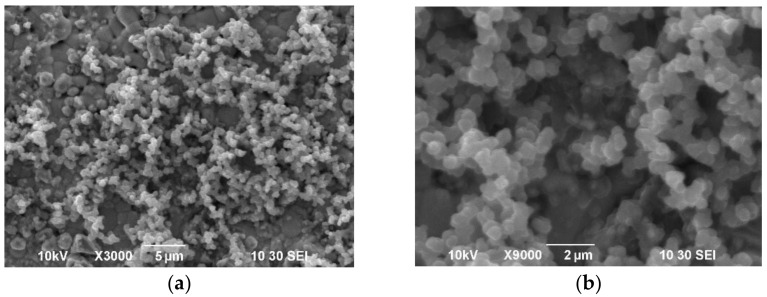
SEM images of the surface of a PPy coating on a non-conductive dense SDC substrate: (**a**) ×3000; (**b**) ×9000.

In this work, a conductive PPy film was synthesized on the surface of the dense SDC substrate by the chemical polymerization of a pyrrole monomer in an aqueous solution of the oxidizing agent ammonium persulfate (NH_4_)_2_S_2_O_8_ and using, in contrast to the previous studies, a salt of p-toluenesulfonic acid from the class of the sodium salts of arylsulfonic acid as a dopant. An aqueous solution containing ammonium persulfate (Sigma–Aldrich, 98 wt.%) with a concentration of 0.03 M and sodium salt of p-toluenesulfonic acid (Sigma–Aldrich, 97.5 wt.%) with a concentration of 0.03 M was prepared, and then pyrrole (Sigma–Aldrich, 98 wt.%) with a concentration of 0.03 M was added under constant stirring at 0 °C. After the beginning of synthesis, the SDC substrate was immersed immediately in the resulting solution and the synthesis of the PPy conductive layer was carried out at room temperature for 3–3.5 h. The sequence of the polypyrrole synthesis process proposed in this study, in which the primary intensive mixing of the reagents was carried out at 0 °C in a solution of distilled water of ammonium persulfate and sodium salt of p-toluenesulfonic acid with the addition of pyrrole monomer, made it possible to obtain a homogeneous reaction mixture, into which immediately after the start of synthesis, the SDC substrate was immersed. SEM images of the surface of the PPy film formed on the SDC substrate are shown in Figure 7a,b. The PPy coating on the SDC surface exhibited chains of submicron particles (~0.5 μm) of a spherical shape, which formed a rather loose conductive coating.

### 3.6. EPD of BCSCuO Barrier Layers on Dense SDC Supporting Substrates with Predeposited PPy Sublayers

**Figure 8:**
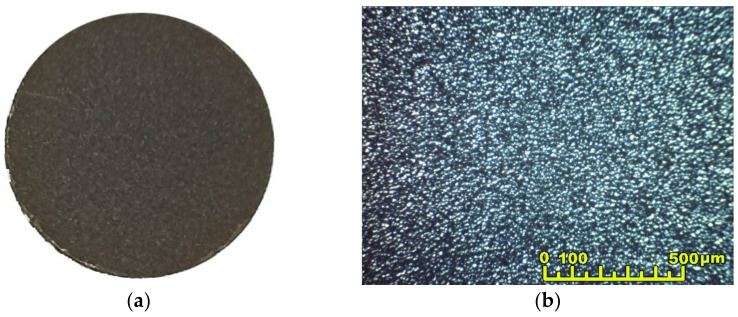
Optical images of the surface of the BCSCuO coating on the dense SDC substrate after deposition using the PPy conductive sublayer followed by drying for 24 h and sintering at 1530 °C for 5 h: (**a**) photograph of the sample; (**b**) optical image.

The electrophoretic deposition of the BCSCuO barrier layer on the dense SDC substrate with the conductive PPy sublayer (substrate thickness of 547 μm, Sample SDC-PPy-BCSCuO_1) was performed from the BCSCuO suspension with a concentration of 10 g/L in the mixed dispersion medium of isopropanol/acetylacetone (70/30 vol.%) with the addition of 0.4 g/L iodine. EPD was carried out under the constant voltage mode U = 80 V for 2.5 min. The layer was dried at room temperature in a Petri dish for 24 h. The thickness of the dried BCSCuO coating was 18.3 µm. The EPD of BCSCuO onto the PPy sublayer was more intense than the deposition onto the Pt sublayer (sample SDC-Pt-BCSCuO). It can be assumed that the deposition onto the platinum sublayer may have been slowed down due to difficulties caused by the electrophoretic infiltration of the deposited particles into the porous structure of the platinum sublayer. The conductivity of both the platinum and PPy sublayers was evaluated to be equal to approximately 500 S/m, while the sheet resistances of the Pt and PPy layers were 200 Ω and 2000 Ω, respectively. Sintering was carried out at a temperature of 1530 °C for 5 h. According to the results of optical microscopy (Figure 8), the BCSCuO layer merged with the substrate, had a bright metallic luster, and no cracks were detected.

For the electrochemical study, we performed the deposition of the BCSCuO barrier layers on both sides of the dense SDC substrate (substrate thickness of 555 μm, sample SDC-PPy-BCSCuO_2). First, the PPy sublayer was formed on one side of the SDC substrate (assumed as the anode side). EPD was performed at a constant voltage of 80 V for 2.5 min (coating thickness of 14 μm), followed by sintering at a temperature of 1530 °C for 5 h. The PPy film was synthesized on the cathode side of the SDC-PPy-BCSCuO_2 sample, then EPD was performed at a constant voltage of 80 V for 1.5 min (coating thickness of 8 μm), followed by sintering at a temperature of 1530 °C for 5 h. The prepared samples SDC-Pt-BCSCuO, SDC-PPy-BCSCuO_1, and SDC-PPy-BCSCuO_2 were used for the electrochemical studies represented in the next section.

### 3.7. Electrochemical Characterization and Microstructure of the Deposited Films

**Figure 9:**
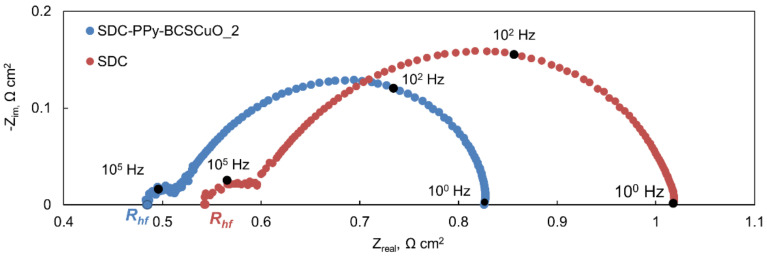
Examples of the spectra obtained at 700 °C under open-circuit conditions for the SDC and SDC-PPy-BCSCuO_2 samples.

The SDC-Pt-BCSCuO, SDC-Ppy-BCSCuO_1, and SDC-PPy-BCSCuO_2 samples and the base SDC sample (550 µm) with deposited Pt electrodes were characterized by the method of impedance spectroscopy in air and under open-circuit conditions, when one of the electrodes was supplied with humidified air, and another one was supplied with humidified hydrogen (3% H_2_O). It should be noted that the Pt electrodes were activated before the measurements (see Section 2.4) to minimize their polarization and, thus, allow the measurement of the ohmic resistance of the samples with/without barrier layers to be as precise as possible. Examples of the spectra obtained are presented in Figure 9. By analyzing the spectra in the ZView-2 program, *R_hf_* was determined, corresponding to the ohmic contribution of the SDC electrolyte (with/without barrier layers). The *R_η_* values, corresponding to the total polarization contribution of the electrodes, were derived from the measurements of the total cell resistance under *dc* current, *R_dc_*, as *R_η_* = *R_dc_* − *R_hf_*. Using these values, the electrolyte resistance and the total polarization resistance of two electrodes were calculated as *R_el_ = S × R_hf_/l* and *R_p_ = S × R_η_*, where *S* is the total effective area of the platinum electrodes on both of the cell sides and *l* is the thickness of the electrolyte substrate.

**Figure 10:**
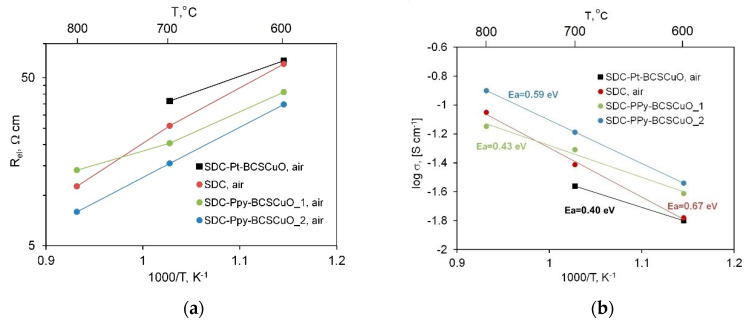
Electrical properties of the SDC samples without/with BCSCuO barrier layers, measured in air by the impedance spectroscopy method: (**a**) Specific ohmic resistance of the samples derived from the spectra; (**b**) Calculated values of the electrolyte substrate conductivity and activation energy.

Figure 10a represents the temperature dependences of *R_el_* obtained under the air atmosphere. Comparing the data obtained for the cells with different configurations, the following patterns can be noted: (1) the resistance of the SDC-PPy-BCSCuO_2 sample with the barrier layers on both sides of the SDC substrate was lower than that of the base SDC and those of the samples with one barrier layer. The drop in the resistance was due to the presence of electronic conductivity in the BCSCuO under the air atmosphere (Section 3.1, Figure 2a); (2) The resistance of the sample with the anode layer deposited onto the Pt sublayer was slightly higher than that deposited through PPy, probably due to the fact that, in this case, the platinum layer prevented Cu diffusion between the BCSCuO layer and the SDC substrate. The Cu-doping of the SDC substrate from the barrier layer can cause p-type electronic conductivity, thus, increasing the total conductivity of the samples; (3) The activation energy of the conductivity in air for the samples with the deposited barrier layers was lower than that of the base SDC, which corresponded to the fact that it was the proton-conducting films that determined the electric properties of the whole cell, despite the small thicknesses of the barrier layers (Figure 10b). The values of the activation energy, E_a_, obtained for the samples with the deposited barrier layers were close to those for the compact BCSCuO sample, which illustrates the determining influence of the deposited films on the whole sample’s behavior. The closeness of the Ea values calculated for the SDC conductivity derived from EIS data to those obtained from the *dc* four-probe measurements (0.67 and 0.70 eV) confirmed the correctness of *R_hf_* determination from the spectra.

**Figure 11:**
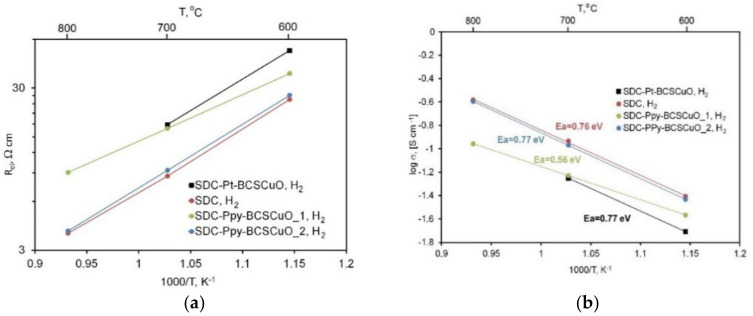
Electrical properties of the SDC samples without/with BCSCuO barrier layers, measured under *OCV* conditions: (**a**) Specific ohmic resistance of the samples derived from the spectra; (**b**) Calculated values of the electrolyte substrate conductivity under the *pO*_2_ gradient (wet air/wet hydrogen) and activation energy.

The resistance of the base SDC sample under the *OCV* conditions (wet hydrogen/wet air, *OCV* (cell sensor) = 1.127 V at 600 °C) (Figure 11a) drastically decreased due to the appearance of electronic conductivity in the SDC electrolyte, which greatly exceeded the ionic conductivity (Table 1). The resistance of all samples with the deposited barrier layers, contrarily, increased, which clearly demonstrated their blocking effect. In this case, the SDC electrolyte from the anode side was protected by the BCSCuO protonic electrolyte that became a unipolar ionic conductor in hydrogen, and its conductivity decreased compared to that in air. The conductivity drop for the SDC-PPy-BCSCuO_2 sample was lower, as the BCSCuO layer on the cathode (air) side to some extent compensated for the increase in resistance on the anode side. The values of activation energy for the conductivity for the samples under *OCV* conditions are shown in Figure 11b. It should be noted that, in contrast to the *dc* four-probe measurements performed under a hydrogen atmosphere (Figure 2a), the activation energy of the total conductivity obtained from the EIS measurement under the *OCV* conditions, when the samples were under the gradient of *pO*_2_, was significantly higher (Figure 11b).

**Figure 12:**
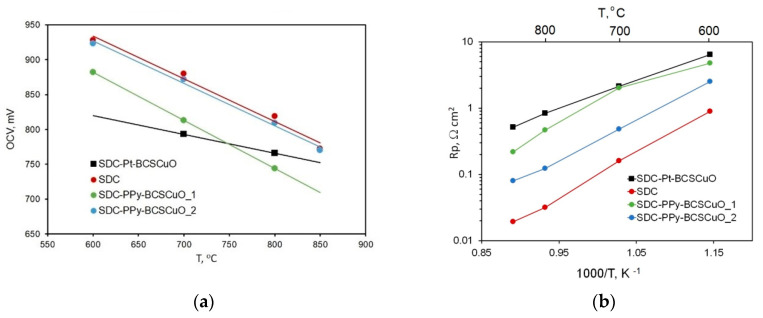
Testing the SDC-based cells with/without barrier layers under OCV conditions: (**a**) OCV values; (**b**) Polarization resistance of the electrodes.

The temperature dependences of the OCV values for the cells under study are shown in Figure 12a. The *OCV* value of the cell with the SDC-PPy-BCSCuO_2 electrolyte was similar to that of the base SDC and even lower for the cells with one barrier layer. Because the BCSCuO barrier layers had a dense sintered structure, a decrease in the obtained OCV values for the samples with barrier layers compared to that of the base SDC could be caused by a combination of such factors as Cu diffusion into the SDC substrate during the high-temperature BCSCuO sintering and the high polarization resistance of the platinum electrodes in contact with the BCSCuO layer. The amount of leakage current can be calculated as follows [61]:

$$I_{e}=\frac{E_{th}-OCV}{R_{el}+R_{p}}$$

where *I_e_* is the electronic leakage current, A/cm^2^; *E_th_* is the theoretical electromotive force according to the Nernst equation [61], V; *OCV* is the open circuit voltage value (measured), V; *R_el_* is the electrolyte resistance to the ionic current, Ω cm^2^; and *R_p_* is the total polarization resistance, Ω cm^2^. For the samples with the deposited barrier layers, the decreases in the electronic leakage current were as follows: for the SDC-PPy-BCSCuO_1 sample, 50% (800 °C) and 41% (700 °C); for the SDC-Pt-BCSCuO sample, 60% (800 °C) and 40% (700 °C); and for the SDC-PPy-BCSCuO_2 sample, 2% (800 °C) and 8% (700 °C). The SDC-Pt-BCSCuO samples had the better results in blocking the electron current. A possible reason for the small increase in the electron leakage current blocking for the SDC-PPy-BCSCuO sample was Cu diffusion from the BCSCuO layer into the SDC substrate, increasing its p-type conductivity. This effect was even more pronounced for the SDC-PPy-BCSCuO_2 sample.

The temperature dependences of the total polarization resistance of the Pt electrodes under OCV conditions are shown in Figure 12b. It can clearly be seen that the polarization resistance of the Pt electrodes for the cells with the barrier layers was much higher than that for the base SDC. This was related to the deteriorating electrochemical activity of the Pt electrodes in contact with proton conductors based on BaCeO_3_ [62]. Replacing Pt electrodes with those more compatible with proton conductors can be an effective way to increase the *OCV* values in the cells with CeO_2_-based electrolyte membranes with deposited proton-conducting barrier layers.

**Figure 13:**
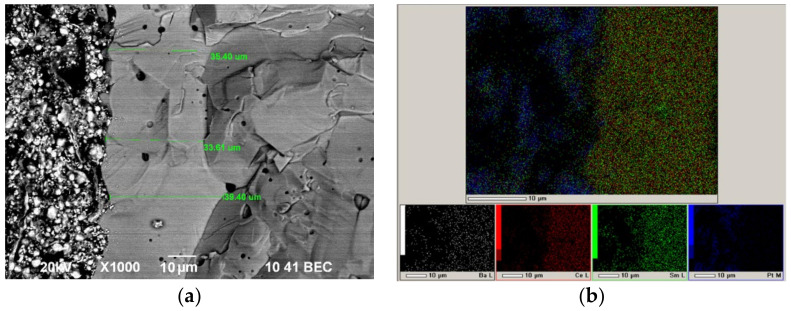
SEM analysis of the cell with the SDC-PPy-BCSCuO_1 electrolyte membrane with Pt electrodes after the measurement cycle: (**a**) Cross-sectional SEM image; (**b**) Integral and individual maps of the element distribution.

To check the barrier layer density, we analyzed the structure of the cell with the SDC-PPy-BCSCuO_1 electrolyte membrane after the measurement cycle. It can be seen (Figure 13) that the BCSCuO anode barrier layer was dense with a few closed pores, and its thickness is even higher (approximately 35 μm) than expected from the deposition weight; therefore, it can ensure efficient protection SDC from the reduction. After the measurements, the cell kept its integrity and no delamination or cracks were observed. Thus, the EPD method through the pre-deposition of the conductive sublayers on the non-conductive electrolyte substrates was validated as an effective and simple-to-implement method for the formation of a dense barrier layer for the SOFC technology.

## 4. Conclusions

In the present work, a comprehensive study was carried out on the formation of barrier layers based on Sm and Cu-doped BaCeO_3_ (BCSCuO) on an SDC-supporting electrolyte by electrophoretic deposition. The formation of the BCSCuO barrier layers with thicknesses within the range of 8–35 μm was performed from non-aqueous suspensions of a micro-sized powder of the BCSCuO electrolyte material, followed by the coating’s drying and sintering at 1500 °C and 1530 °C. As the zeta potential value of the initial BCSCuO suspension was relatively low (+11 mV), the suspension was modified by the addition of molecular iodine (0.4 g/L) to increase its conductivity. Despite the fact that the zeta potential did not change after the iodine addition, this additive facilitated deposition. To carry out EPD on the non-conductive SDC substrates, the preliminary formation of a conductive sublayer of finely dispersed platinum (by a drop method) or polypyrrole (by the chemical polymerization of pyrrole in an aqueous solution) on their surface was carried out. A feature of the PPy film synthesis technology proposed in this study was the use of an increased concentration of reagents and intensive mixing of the aqueous solution of the reaction mixture immediately before the SDC substrate was immersed in it. It was shown that the Pt sublayer could be successfully used for the cyclic EPD, while the PPy conductive layer required deposition in one step due to its burning out after the layer sintering. The samples with one BCSCuO barrier layer (on the anode side) deposited using Pt coating (SDC-Pt-BCSCuO) and PPy coating (SDC-PPy-BCSCuO_1) and with two BCSCuO barrier layers (on the anode and cathode sides; SDC-PPy-BCSCuO_2) were supplied with Pt electrodes and tested in air and under *OCV* conditions. The data obtained in air demonstrated that the technology used to form a conductive sublayer had a significant effect on the conductivity value of the BCSCuO barrier layer. Namely, the sample obtained by the cyclic EPD method on the Pt sublayer had a lower electrical conductivity compared to the sample obtained in one EPD cycle on the PPy sublayer. This may be due to the loose nature of the Pt coating, which may reduce the effective density of the deposited BCSCuO electrolyte. It was found that the effect of blocking the electronic current in the SDC substrate under *OCV* conditions was maximal for the cells with barrier layers deposited on the anode side: for the SDC-PPy-BCSCuO_1 sample, 50% (800 °C) and 41% (700 °C); and for the SDC-Pt-BCSCuO sample, 60% (800 °C) and 40% (700 °C). Despite the high performance of the cell with two barrier layers SDC-PPy-BCSCuO_2, the blocking effect was significantly lower and reached only 2% (800 °C) and 8% (700 °C). The polarization resistance of Pt electrodes had a significant effect on the decrease in the *OCV* value of the cells with BCSCuO barrier layers, which necessitates the selection of other electrodes that are electrochemically compatible with this electrolyte for future studies. The performed study confirms the applicability of the EPD technology for the formation of dense barrier layers of doped BaCeO_3_ in the fabrication of SOFCs with CeO_2_-based supporting electrolytes.

## Figures and Tables

**Table 1:** The values of the partial and total conductivities for the BCSCuO and SDC compacts at different temperature and *pO*_2_ conditions.

Samples	600 °C			700 °C		
	σ_e_, mS/cm	σ_i_, mS/cm	σ_tot_, mS/cm	σ_e_, mS/cm	σ_i_, mS/cm	σ_tot_, mS/cm
**0.21 atm**						
BCSCuO	1.6	7.2	8.8	6.4	10.3	16.7
SDC	~0.01	6.1	6.1	~0.01	18.4	18.4
**10^−22^ atm**						
BCSCuO	0.07	6.9	7.0	0.07	9.6	9.7
SDC	8.1	6.1	14.09	201.9	18.4	220.3

## Data Availability

Not applicable.
